# OPTiM: Optical projection tomography integrated microscope using open-source hardware and software

**DOI:** 10.1371/journal.pone.0180309

**Published:** 2017-07-11

**Authors:** Thomas Watson, Natalie Andrews, Samuel Davis, Laurence Bugeon, Margaret D. Dallman, James McGinty

**Affiliations:** 1 Photonics Group, Department of Physics, Imperial College, London, United Kingdom; 2 Department of Life Sciences, Imperial College, London, United Kingdom; University of California Berkeley, UNITED STATES

## Abstract

We describe the implementation of an OPT plate to perform optical projection tomography (OPT) on a commercial wide-field inverted microscope, using our open-source hardware and software. The OPT plate includes a tilt adjustment for alignment and a stepper motor for sample rotation as required by standard projection tomography. Depending on magnification requirements, three methods of performing OPT are detailed using this adaptor plate: a conventional direct OPT method requiring only the addition of a limiting aperture behind the objective lens; an external optical-relay method allowing conventional OPT to be performed at magnifications >4x; a remote focal scanning and region-of-interest method for improved spatial resolution OPT (up to ~1.6 μm). All three methods use the microscope’s existing incoherent light source (i.e. arc-lamp) and all of its inherent functionality is maintained for day-to-day use. OPT acquisitions are performed on *in vivo* zebrafish embryos to demonstrate the implementations’ viability.

## Introduction

In biological and biomedical research there is an increasing trend towards 3D cell cultures and *in vivo* model organisms to provide more physiologically relevant context. This requires the development of novel 3D optical imaging techniques for mesoscopic samples (~mm dimensions) that can potentially provide the functional information obtainable using established quantitative microscopy techniques applied to traditional 2D cell cultures. However, commercial instrumentation capable of 3D optical imaging (e.g. confocal microscopy, etc) is expensive and often only available through central imaging facilities, limiting accessibility. Conversely, standard wide-field microscopes are more widely available and many research labs have their own microscope(s). In this paper we describe a simple and inexpensive open-source adaptor that sits in a standard microscope stage, providing the necessary alignment and sample rotation required for optical projection tomography (OPT) [1], allowing 3D reconstructions from transmitted light and/or fluorescence acquisitions of ~mm sized samples using the microscope’s incoherent light sources (e.g. arc-lamp). Open access OPT has been proposed previously [2,3], but requires a fully custom-built OPT system. Our design optimises the use of available components and lab space, by adapting an existing microscope using the OPT plate. The plate can easily be removed allowing conventional 2D wide field imaging.

OPT is often described as the optical equivalent of X-ray computerized tomography. Briefly, in OPT a series of wide field in focus images, or ‘projections’, are recorded as a sample is rotated. These in focus projections can be acquired by reducing the numerical aperture (NA) of the imaging system such that the depth of field is comparable to the axial extent of the sample (this will be referred to as ‘standard’ OPT) [1,4] or by scanning the focal plane through the sample at each projection angle such that in-focus information is acquired from the sample’s full axial extent (focal scanning OPT) [5]. In both approaches if the imaging system is telecentric (i.e. the lateral magnification is constant along the optical axis over the extent of the sample) then the projection image can be considered a parallel projection and a filtered back projection (FBP) algorithm used for 3D reconstruction [6]. Furthermore, if the axis of rotation (AoR) about which the sample rotates is aligned correctly, each row of pixels on the camera acquires information from a single slice through the object at every projection angle, allowing each cross-sectional slice to be reconstructed independently, simplifying the implementation of the FBP algorithm.

Using this adaptor, we describe three implementations of OPT on an inverted microscope. The first and most direct implementation works at low magnification (×4 and below) and simply requires the addition of an aperture directly behind the objective to extend the depth of field for standard OPT. At higher magnifications placing the aperture directly behind the objective leads to measurable non-telecentric performance. Therefore the second implementation uses an image relay attached to the camera port of the microscope such that the limiting aperture can be positioned conjugate to the pupil plane, allowing standard OPT to be performed at higher magnifications. The third system makes use of the full NA of the objective lens by replacing the final element of an image relay with an electrically tuneable lens (ETL). Oscillating the focal length of the ETL performs remote focal scanning OPT (RFS-OPT) [7], resulting in improved light‑collection efficiency and reconstruction spatial resolution by imaging with the full NA of the objective lens.

## Open-source OPT plate

**Fig 1:**
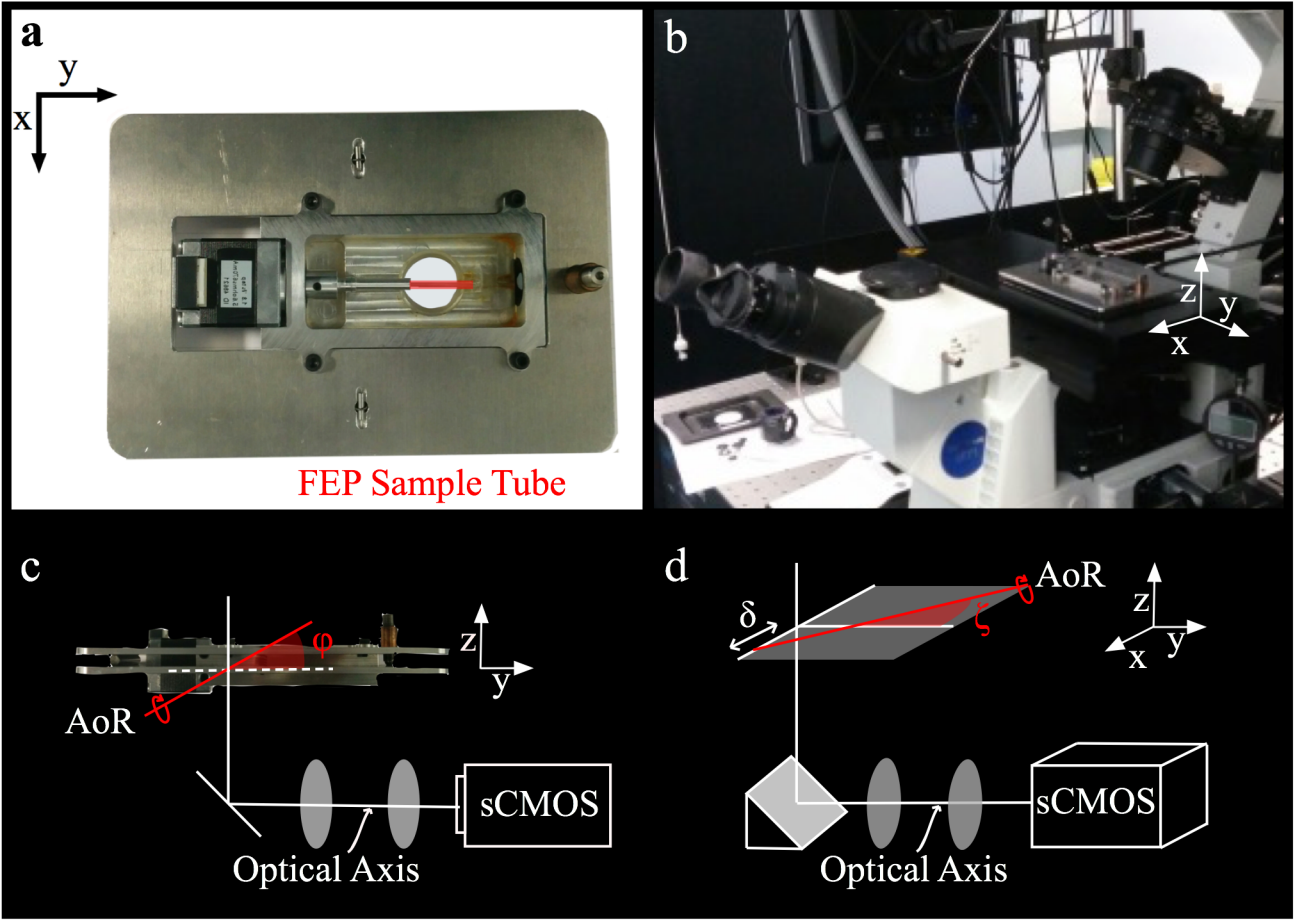
(a) Photograph of the OPT plate detailing the stepper motor, water-tight chamber and position of the sample tube (shown in red). (b) Photograph of an inverted microscope adapted for OPT, with the plate inserted in the microscope stage aperture. (c) The custom chamber is suspended from an upper plate, with the lower plate seated in microscope stage. For alignment purposes, the axis of rotation (red line) is adjusted until it is orthogonal to the optical axis, setting the tilt angle φ = 0°. (d) In addition the axis of rotation should be approximately centred on the camera sensor and rotated to align with the sensor pixels, setting the shift δ ≈ 0 and rotation angle ζ ≈ 0°.

Fig 1(A) shows photographs of the custom aluminium OPT plate fabricated for an inverted microscope. Further technical information and CAD drawings are available in the Supplementary Material and (http://www.imperial.ac.uk/photonics/research/biophotonics/instruments—software/optical-projection-tomography-opt/opt-microscope-adaptor-plate/). A stepper motor (NM08AS-T4 and A-MCA-PMF3, Laser 2000 Ltd) and water chamber were attached to the upper aluminium plate, which in turn was mounted on a lower plate with a hinge, fine adjustment thread and tension springs to allow the tilt angle of the motor’s AoR to be adjusted. This lower plate was mounted securely into the microscope stage insert aperture (160 × 110 mm). The stepper motor axle passed through a rubber O-ring port in the chamber wall and a mounting-port converted the 5 mm motor axle to a 1.6 mm port to hold the fluorinated ethylene propylene (FEP) tube in which samples were mounted. During an *in vivo* acquisition the chamber was filled with water to produce a refractive index matched environment. Simple modifications to the chamber design would allow a variety of tube diameters, and therefore sample sizes, to be mounted and imaged. In the work described here samples were mounted in the same size FEP tubing, resulting in an effective sample diameter of ~1 mm. All acquisitions described were therefore optimised for this sample size (e.g. magnification, effective numerical aperture, etc). Fig 1(B) shows the sample chamber in place on a commercial microscope (IX-71, Olympus UK Ltd). Importantly, the microscope retains all its imaging and illumination capabilities so can still be used for day-to-day imaging tasks as required.

The overall cost of the OPT plate is ~£1000; ~£800 for the commercial components (stepper motor, motor controller, bearings, etc) at the time of purchase and ~£200 for custom manufactured aluminium plates and chamber.

The alignment requirements for OPT are illustrated in Fig 1C and 1D and relate to the relative position and orientation of the AoR to the optical axis. The red line represents the AoR around which the sample rotates during an acquisition. To permit the independent reconstruction of cross‑sectional slices, the AoR must be orthogonal to the optical axis of the imaging system. Therefore in Fig 1(C) the tilt angle has to be adjusted such that φ = 0. This degree of freedom must be provided by the OPT plate itself. Fig 1(D) illustrates a relative rotation of the AoR with respect to the vertical (as defined by the pixel columns in the camera) and a lateral shift with respect to the centre of the field of view. These misalignments can be minimized by translation of the microscope stage and physical rotation of the camera on its port before data is acquired or can be corrected post-acquisition before reconstruction. In either case the OPT plate does not require adjustments to correct for these degrees of freedom. A calibration and alignment procedure are detailed in the Supplementary Material.

## Low magnification OPT

**Fig 2:**
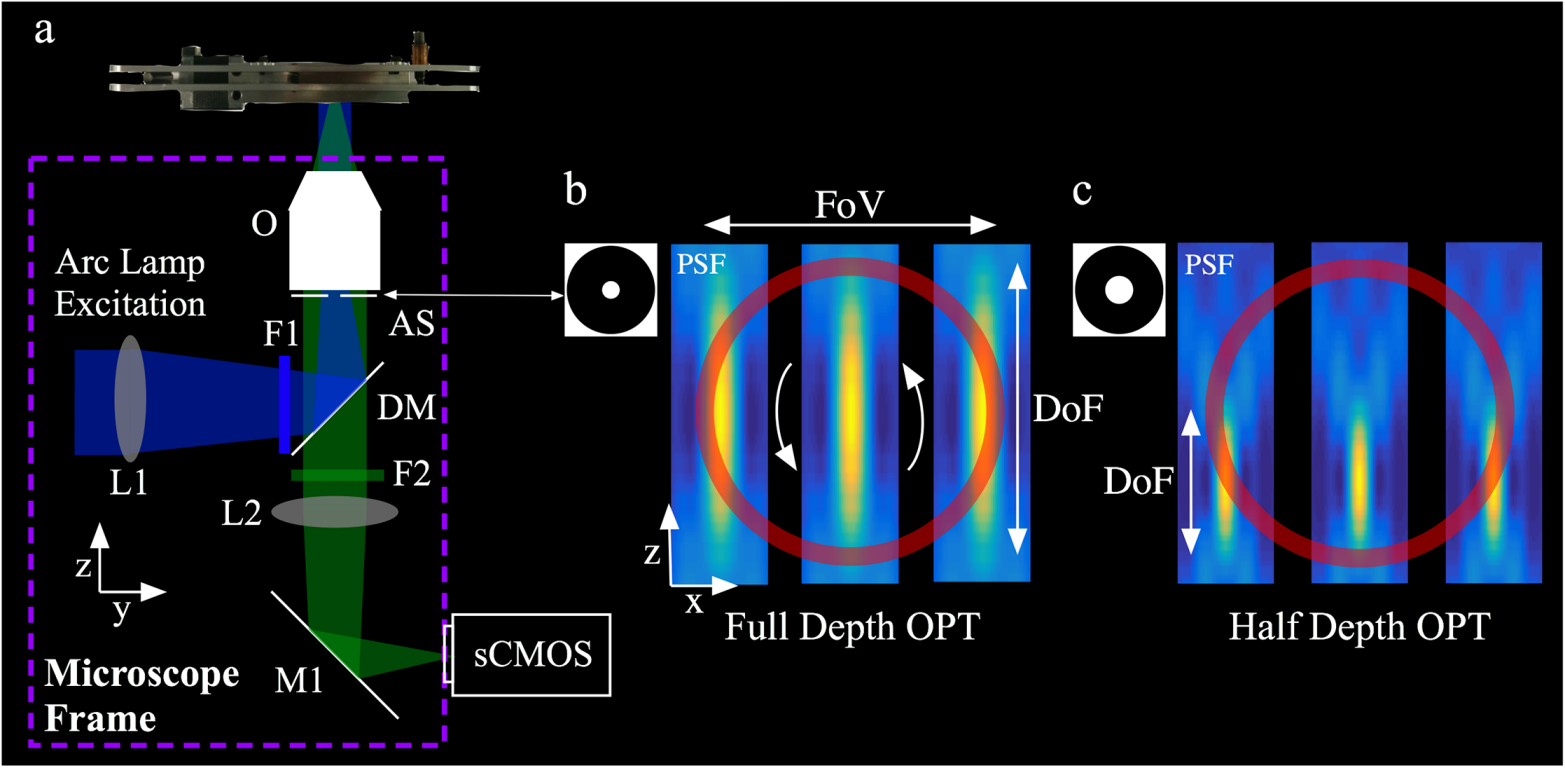
(a) Schematic showing a microscope adapted for OPT. The only additional component inside the microscope (indicated by the dashed box) was the aperture placed directly behind the objective lens to reduce the NA. Simulated shift invariant axial PSF for (b) full-depth OPT, and (c) half-depth OPT. The sample cross section is indicated by red circle. Note that the PSFs are scaled for illustrative purposes.

At low magnification (×4 and below), the effective NA of the microscope can be reduced by placing an aperture directly behind the objective lens, as illustrated in Fig 2(A), without causing measurable non-telecentric performance. Low magnification imaging was performed with a ×4 objective with an intrinsic 0.13 NA (UPLANFL 4x, Olympus Ltd). For OPT, if the focal plane is positioned at the AoR, the NA must be reduced such that the depth of field covers the whole sample (i.e. ~1 mm) and an acquisition only needs a 180° rotational scan. This required an aperture of ~2.3 mm to produce an effective NA ~0.025. If the focal plane is positioned in front of the AoR, the relative NA can be increased by a factor of $\sqrt{2}$ such that the depth of field covers the front half of the sample, leading to improved light collection and diffraction-limited spatial resolution, but requiring a 360° rotational scan–this represents the most common experimental configuration for OPT. Fig 2B and 2C show simulated effective axial point spread functions (PSF) in the case of full- and half-depth of field acquisitions respectively, the red circle indicates the cross‑sectional circumference of the sample. The fact that the shifted PSFs are parallel illustrates that the system is telecentric over the extent of the sample.

**Fig 3:**
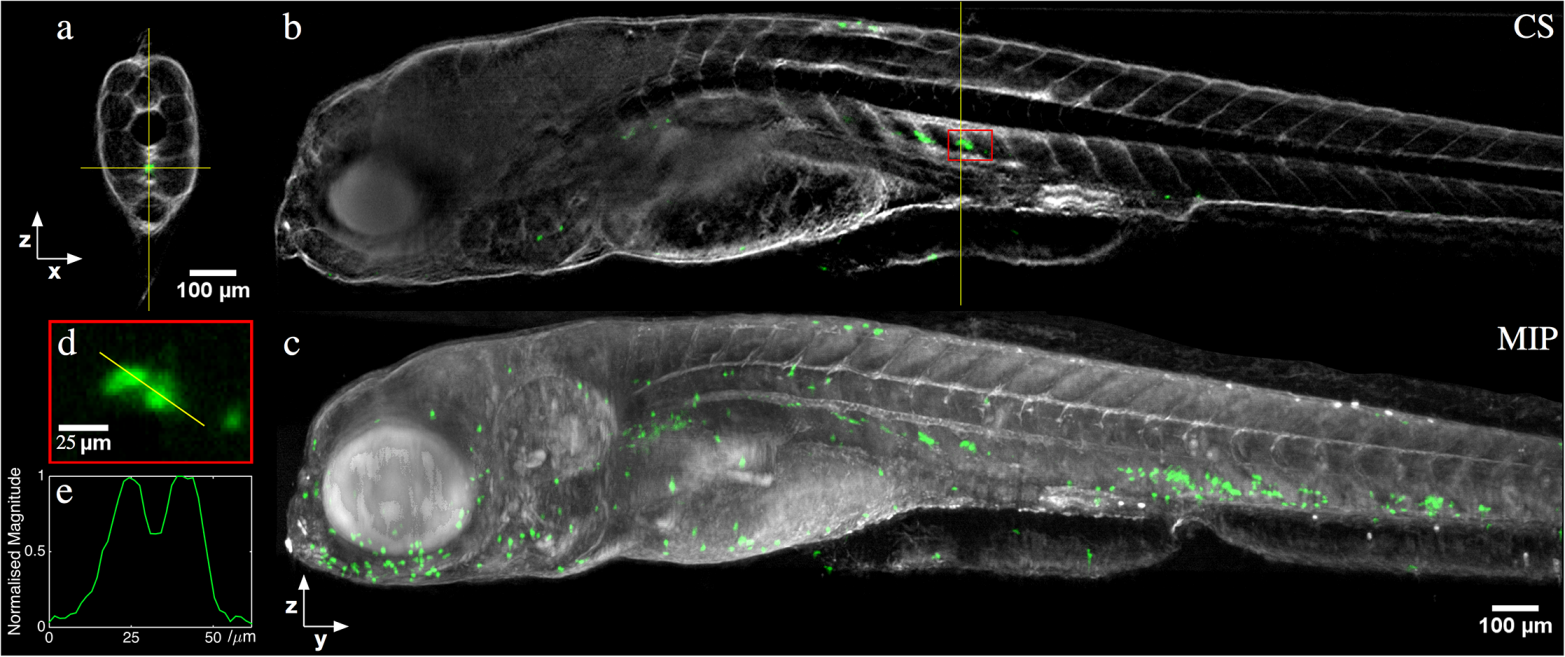
*In vivo* half-depth of field OPT reconstruction of a 5 days post fertilization transgenic mpx:GFP zebrafish, combining sequential fluorescence (for neutrophil GFP expression, shown in green) and transmission (for zebrafish morphology, shown in grey) acquisitions. (a) Single slice through the reconstruction, accompanying (b) YZ cross-section (CS) along vertical yellow line in (a). (c) Maximum intensity projection (MIP) through entire reconstructed volume. (d) Magnified view of reconstruction within red box indicated in (b), and (e) line profile through neutrophils cells.

Fig 3 shows the combined fluorescence and transmitted light reconstructions from an *in vivo* acquisition performed on a 5 days post fertilization Tg(mpx:GFP) zebrafish embryo. Using the half-depth of field OPT approach, 400 projection images were acquired for both fluorescence and transmitted light at 0.5 s integration time per image, resulting in a total acquisition time per channel of 260 s. To produce an effective NA ~0.055, an aperture of 5 mm was used. Disks with the required diameter and aperture size were fabricated from black nylon (282–0676, RS Components Ltd). In this configuration, reconstructions had an isotropic spatial resolution of 5.6 ± 0.4 μm (see Supplementary Material).

Fig 3(A) shows a reconstructed slice through the zebrafish (i.e. an XZ slice, c.f. Figs 1 and 2), with the fluorescence reconstruction in green and transmitted light in grey. Fig 3(B) shows the YZ slice for the position indicated by the vertical crosshair in (a), while (c) is a maximum intensity projection through the whole volume. Fig 3D and 3E show a region from the fluorescence reconstruction (indicated by the red box in 3(c)) of two neutrophils in close proximity and an intensity line plot through them respectively. 3D visualisation is available as S1 Video (VLC can be used to play the.avi files).

The zebrafish used in this study were maintained according to standard practices and all procedures conformed to UK Home Office requirements (ASPA 1986 under the project licence PPL 70/6655). This project licence and the work carried out under it was reviewed and approved by the Animal Welfare and Ethics Committee (AWERB), Imperial College London.

In the standard approach to OPT described above, the only computer-controlled components are the stepper motor and camera. Therefore we have developed a simple acquisition program implemented in μManager [8] to control these components (Zaber Ltd NM08AS-T4, Andor Ltd Zyla 5.5). Download instructions are available from, (http://www.imperial.ac.uk/photonics/research/biophotonics/instruments—software/optical-projection-tomography-opt/opt-microscope-adaptor-plate/)).

Adapting this program for other motors and cameras supported by μManager is relatively straightforward. The total reconstruction time for a volume of 760×760×2160 pixels was <5 minutes (physical size 1.2×1.2×3.5 mm) using the GPU-accelerated ‘iradon’ function in MATLAB (The Mathworks Inc.). Alternate open-resource reconstruction software are available as ImageJ plugins [9,10], and the reconstruction procedure is described in [9] (supplementary material).

In comparison to alternative 3D imaging techniques, laser scanning confocal microscopy (LSCM) would typically take between 6–20 minutes (dependant on sample luminance) [11] to image an identical sample volume as shown above, while a commercial light sheet microscope (LSM) may take between 2–4 minutes [12] (OPT ~ 4 minutes). The achievable lateral resolution of LSMC and LSM systems are greater than the equivalent OPT counterparts as they do not have to reduce the NA in order to extend the DoF, but their axial resolution are several times the lateral resolution [13]. The absolute reduction in resolution between OPT and the alternative techniques will depend of the size of the sample, relative to the objective NA. However, while LSM and LCSM may provide improved resolving capabilities, the cost of purchasing a new laser scanning confocal or light sheet microscope is significantly more than outfitting an existing wide-field microscope for OPT.

For low magnifications this standard approach to OPT is relatively simple to implement, requiring only the addition of the OPT plate and an aperture directly behind the objective to reduce the effective NA. Additionally the alignment requirements can tolerate errors in the stepper motor motion and small errors in the stage angle, allowing a relatively quick alignment procedure. Importantly, the microscope retains all its imaging and illumination capabilities so can still be used for day-to-day imaging tasks as required.

## High magnification OPT

**Fig 4:**
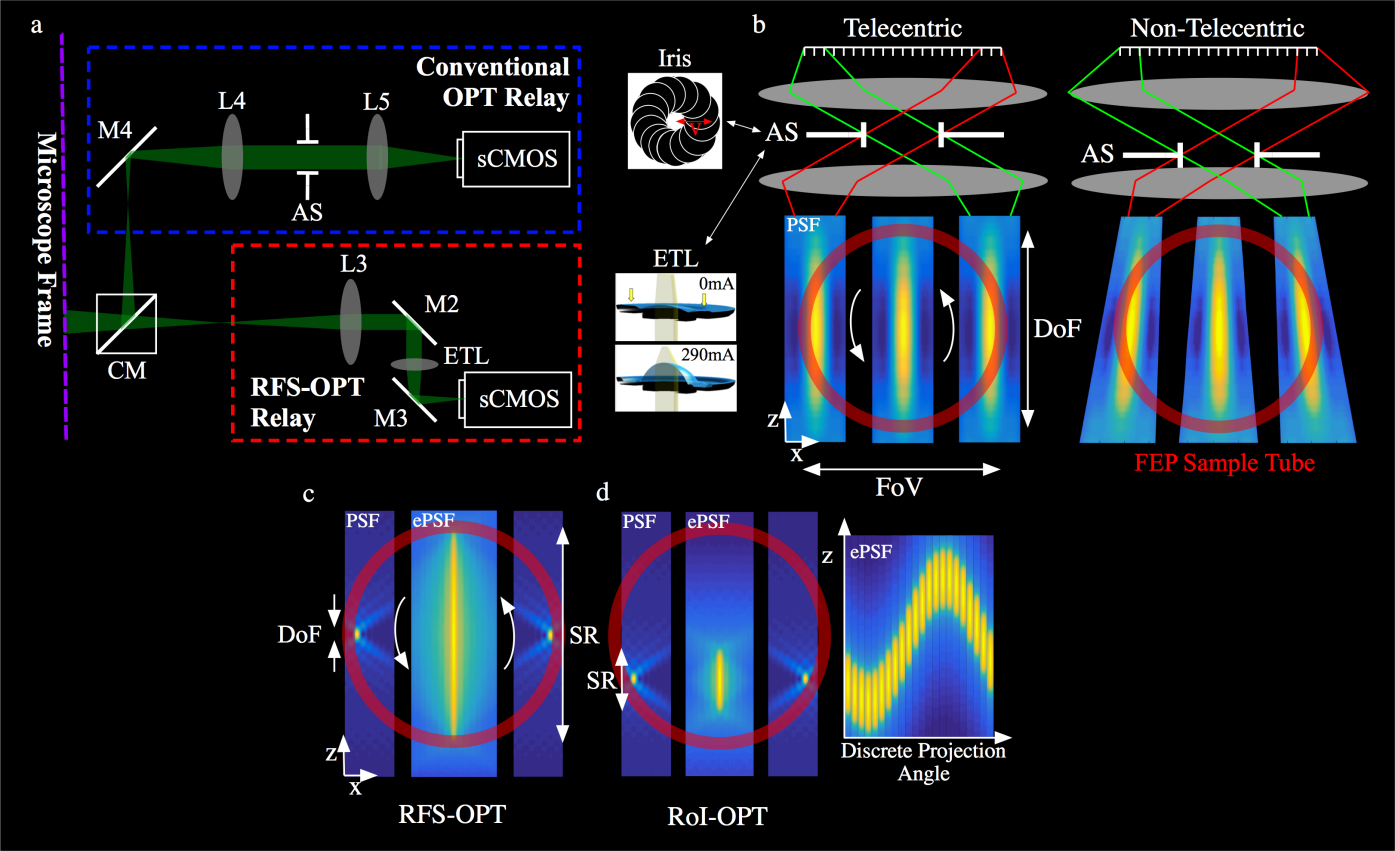
(a) External relay added to the camera port of the microscope for higher magnification standard OPT and remote focal scanning OPT (RFS). A continuation of the components within the microscope frame (Fig 1(C)), CM removable cube mirror. External RFS-OPT relay (red box): L3 achromatic doublet, M2/M3 mirror cubes, ETL electrically tunable lens. Conventional OPT relay (blue box): M4 mirror, L4/L5 achromatic doublets, AS variable iris acting as aperture stop. (b) The variable iris or ETL act as the aperture stop in the conventional or RFS OPT systems respectively. The size of the iris or scan range of the tunable lens determine the respective DoF, but axial displacement away from the conjugate pupil plane can lead to non-telecentric performance. The 3D axial PSF is no longer shift invariant, resulting in a depth dependant magnification. Note the point spread functions are scaled for illustrative purposes. (c,d) Simulation showing the static axial PSF (exterior panels) and effective axial PSF(central panels) for RFS-OPT and region of interest (RoI) OPT. (c) RFS-OPT with a scan range covering the full axial extent (d) RoI-OPT uses a smaller SR to increase the contrast to noise ratio over a desired region of interest. The RoI is tracked in depth, by adjusting the focal offset required to track the RoI during an acquisition (i.e as the sample rotates to each projections angle).

The primary pupil plane (also called the Fourier plane) of an objective lens is typically located within the objective housing and is the position a limiting aperture should be placed to ensure telecentric performance. Therefore, positioning an aperture directly behind an objective, while reducing the NA, leads to non-telecentric performance due to the axial displacement between the pupil plane and limiting aperture. While this non-telecentric behaviour is not observable for magnifications of ×4 and below, at higher magnifications the imaging process cannot be considered a parallel projection. In order to access the Fourier plane, an external relay was added to the camera port of the microscope, composed of two achromatic doublets with focal lengths of 300 and 200 mm (AC508-300-A-ML and AC508-200-A-ML, Thorlabs Ltd) and an iris (variable aperture) placed in a conjugate plane to the objective lens’ pupil to control the NA (conventional OPT relay blue box Fig 4(A)). Using a ×20 objective (UPLANFL 20x, Olympus Ltd), the net magnification of this setup was ~12.6x, with a field of view of ~1.3 mm.

Fig 4(B) is a schematic showing the transition from telecentric to non-telecentric performance by axial displacement of the limiting aperture. The cross-sections through the simulated PSFs indicate how the non-telecentric system has an axially dependent magnification (i.e. the 3D PSF is no longer shift invariant) leading to a breakdown in the parallel projection assumption. While the addition of the image relay makes this implementation of OPT slightly more complicated, it retains telecentricity for all objective magnifications. An additional benefit is that there is no reduction in the illumination NA. This is important when using an incoherent light source (e.g. an arc-lamp) since it avoids a potential reduction in illumination efficiency due to a limiting aperture positioned directly behind the objective lens. Importantly, by simply removing/opening the aperture in the image relay the microscope can be used at full NA for routine laboratory applications.

**Fig 5:**
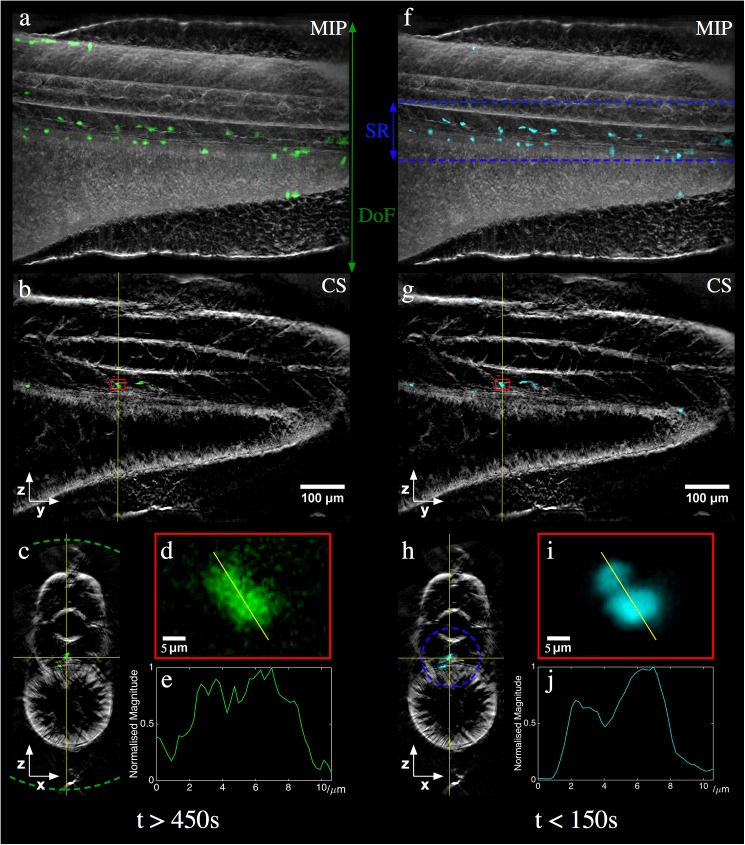
Fluorescence (green/cyan) and transmitted light (grey) reconstructions from an *in vivo* acquisition of a 5 dpf Tg(mpx:GFP) zebrafish using (a-e) an image relay for standard full-depth of field OPT at NA~0.035 and (f-j) RoI-OPT at full NA with an axial scan range (SR) of 130 μm. (a,f) MIP of full reconstruction, (b,g) single YZ slice, (c,h) single XZ slice with depth of field and SR respectively indicated by dotted lines, (d,i) fluorescence reconstruction from region indicated by red box and (e,j) corresponding intensity line profile.

Fig 5A–5E show different representations of the fluorescence and transmission reconstructions from a full-depth of field set-up using the image relay from an *in vivo* acquisition of a Tg(mpx:GFP) zebrafish embryo. An aperture of ~1 mm was used to reduce the effective NA to ~0.035, with an integration time of 1 s per projection leading to a total acquisition time greater than 450 s per channel. 3D visualisation available as S2 Video.

High magnification OPT is best suited for smaller samples, in order to maximise resolution. At 4x magnification, the optical system is pixel-limited for samples <300μm in extent, and as such high magnification OPT is recommended to achieve diffraction limited images.

## Remote focal scanning OPT

The main trade-off in the imaging performance of OPT results from matching the depth of field to the ~radius of the sample, which requires a reduction in NA and therefore the achievable diffraction limited lateral resolution. This trade-off can be mitigated by scanning the focal plane of a high NA objective through the sample at each projection angle [5,14,15]; a remote focal scanning (RFS-) implementation using an electrically tuneable lens (ETL) was recently demonstrated by the authors on a bespoke imaging system [7]. The image relay described was adapted to perform RFS-OPT on a commercial microscope, as shown in the red box in Fig 4(A). In this system the image relay was comprised of a 180 mm focal length achromatic doublet (AC508-180-A-ML, Thorlabs Ltd) and an ETL (EL-10-30, Optotune Ltd) positioned at a conjugate plane to the primary pupil to provide telecentric focal scanning. The ETL can provide both a static focal plane position by applying a constant current and an oscillating focal plane position by applying a time-varying current. The clear aperture of the ETL was larger than the image of the objective’s pupil and therefore did not reduce the NA of the ×20 objective (NA~0.4).

Fig 4(C) illustrates the effective PSF (central panel) for the typical approach to focal scanning OPT, where the focal plane was scanned over the full axial extent of the sample (red circle). Therefore in-focus information from the whole sample was acquired at every projection angle as well as out-of-focus light. Fig 4(B) shows an alternative scanning approach, demonstrated here for the first time in OPT to our knowledge, where the axial extent of the scan range was limited to a region of interest (RoI). During a RoI-OPT acquisition the scan range (SR) was kept constant, but an axial offset was applied to the ETL (i.e. an additional constant current) to track the RoI as it rotated. The resultant sinusoidal offset of the SR over a full acquisition is shown in Fig 4(D).

Fig 5F–5J shows the reconstruction for a RoI-OPT acquisition of the same zebrafish shown in 5(a-e). 3D visualisation available as S3 Video. A sinusoidal current of amplitude 80 mA at 10 Hz was applied to produce a scan range of 130 μm for RoI-OPT of the zebrafish trunk axial vessels. 400 projections were acquired with an integration time of 0.2 s, resulting in a total acquisition time of ~150 s per channel. The increased imaging NA significantly improves the overall light collection efficiency of RFS/RoI-OPT compared to the standard OPT approach as well as the lateral spatial resolution per projection image for the in-focus light. This provides improved spatial resolution and contrast‑to‑noise ratio in the reconstruction (illustrated by the improved discrimination between adjacent cells shown in Fig 5I and 5J) at a reduced acquisition time and therefore excitation light dose. There is additional discussion about the relative performance of these different acquisition approaches in the Supplementary Material. By applying a constant current to the ETL, again the microscope can still be used at full NA for routine laboratory applications.

RFS- and RoI-OPT overcome the trade-off between sample size and resolution that exists in standard OPT. As a consequence, the theoretical resolution of these OPT systems is similar to LSM and LCSM. In a practical environment, the resolution is mechanically limited by rotation stability, and sample limited due to the shape of the optical transfer functions (see supplementary material).

## Conclusion

As the use of mesoscopic 3D biological and biomedical samples increases, there is a need to provide efficacious and cost-effective imaging instrumentation. The current commercial solutions (e.g. confocal microscopy) are expensive and are therefore not part of the imaging capabilities within most biological/biomedical research groups, but rather are available through central imaging facilities if at all. As a possible solution to provide cost-effective and readily available 3D imaging, we have designed an open-source OPT plate that can be integrated into a commercial inverted wide‑field microscope, with hardware and software made freely available. The plate, which slots into a standard microscope stage, incorporates a stepper motor for sample rotation and a tilt adjustment to ensure that the axis of rotation is perpendicular to the optical axis.

The most straightforward approach for OPT using this plate was implemented at low magnification (×4 and below) by simply placing an aperture directly behind the objective to reduce its NA and therefore increase the depth of field. To perform OPT at high magnifications while retaining telecentric performance, which is required to ensure the parallel projection assumption, an external image relay and limiting aperture were added to the camera port of the microscope. Finally, to improve the spatial resolution and light collection efficiency, the relay system was adapted to include an electrically tuneable lens to perform remote focal scanning (RFS-OPT), including an alternative acquisition approach at a reduced scan range that tracked a specific region of interest (RoI-OPT). These different approaches to OPT on a commercial wide-field microscope were provided to demonstrate how a system can be developed in a modular way as the imaging requirements change (e.g. from low to high magnification). Importantly, all intrinsic functionality of the microscope was retained and available for routine use in the laboratory under all three implementations.

## Supporting information

S1 File Supplementary information. (DOCX)

S1 Fig CAD rendering of the OTP adaptor. View of (a) the top plate, (b) the bottom plate and (c) sample chamber, stepper motor and axle adaptor in which the tube containing the sample is inserted and held.
(TIFF)

S2 Fig Diagram of calibration procedure for real data of fluorescent microspheres (200nm diameter), 4x magnification using system from Fig 1C. (a) Raw projection from full-DoF OPT acquisition. (b) The spheres are found from the raw projections, and their trajectories recorded using simple peak finding MATLAB software (colour represents the same sphere through figs b-f). (c) The mean value of the recorded x-positions provides the axis of rotation (AoR) horizontal shift, δ from sensor centre, for each z-position. Applying a linear fit, gives the AoR rotation angle ζ. These values are used before reconstruction to shift and rotate the raw projections. (d) Sinusoid fitted to the recorded x-positions for all sphere traces. (e) Recorded y-positions of the microspheres. If all spheres have the same amplitude of variation over the acquisition cycle, this suggest there exists a tilt in the system, φ, and the custom stage angle will need to be altered. If the spheres y-deviation varies across the FoV, this suggest the system is not telecentric, and an external aperture and relay system may be required (only necessary for >4x magnification). (f) Difference between recorded x-positions and fitted sinusoid. Compare deviations in (e-f) with the diffraction limit of this system is ~4.5μm / 3 pixels (reduced NA~.055). As these effects are significantly smaller than the diffraction limit, the impact on reconstruction quality will be negligible.
(TIFF)

S3 Fig Data acquired using 20x objective, in an external relay setup. (a) Example of a bean sinogram of a 4 μm fluorescent microsphere with associated fitted sinusoid (dotted line). (b) Measured AoR motion for different acquisition modes. Note the additional RFS trace represents a second independent acquisition demonstrating the repeatability of the motion. Single slice reconstructions of 4 μm fluorescent microspheres (c) without and (d) with motor motion correction applied.
(TIFF)

S4 Fig Example of change in optical transfer function (OTF), when implanting focal scanning with different scan ranges (SR). Simulations modelled in MATLAB for 20x, 0.4NA objective, focusing into 3mm of water. (a) 2D representation OTF of RFS-OPT with a scan range of ~65μm (40mA current modulation). (b) RFS-OPT at maximum scan range of ~450μm (290mA current modulation). (c) Example of static OTFs at increasing focus depths. (d) Line profiles across centre of OTF, plotted on log scale, normalized to integrate to 1. Spatial frequency is normalized to the cut-off frequency for an NA of 0.4. Also shown are the diffraction limited (DL) profiles for an NA~0.4, which is the full NA used in the scanning procedure, and a reduced NA~0.03, which is equivalent to a depth of field ~450μm.
(TIFF)

S5 Fig Comparison between the different OPT techniques, looking at a raw projection of >200nm fluorescent microspheres. (a) Conventional OPT with the DOF covering the whole sample, NA~0.025. (b) Conventional OPT with the DOF covering the front half of the sample. (c) RFS-OPT at maximum scan range. (d) RoI-OPT tracking a region of width ~65μm. The dashed-red circle represents the object used to perform the pre-scan procedure, and lies with the region of interest for RoI-OPT.
(TIFF)

S6 Fig Cross sections (CS) through reconstructed volume of 200nm fluorescent microspheres. (a) Resolution measurements on reconstructed slices of fluorescent microsphere sample. Note the spheres depicted in (a-c) are not the same, but are representative of the smallest object reconstructed. (a) Half DoF OPT, NA ~0.05 (full DoF OPT not represented as the resolution was significantly worse). (b) RFS-OPT with ~400 μm scan range and NA ~0.4. (c) RoI-OPT with scan range reduced to ~65 μm and NA ~0.4. (d) Gaussian fits and raw data from the line profiles shown in (a-c) showing increase in light collection efficiency. (e) Normalised gaussian fitting to illustrate resolution improvement from conventional to RFS systems. (f-h) Reconstructed slice through a sample of 200 nm fluorescent microspheres for (f) Half-DoF OPT (and Media 5), (g) RFS-OPT and (h) RoI‑OPT (and Media 6). ETL scan range shown by red circle. Note that colour scales are non-linear to display both bright and faint objects. (i-k) Magnified view of region of interest (yellow box), highlighting the improvement in CNR and reduction of streak artefacts within the region of interest with RoI-OPT (with linear colour scales).
(TIFF)

S1 Video 3D visualisation video of *in vivo* half-depth of field OPT reconstruction of a 5 days post fertilization transgenic mpx:GFP zebrafish, combining sequential fluorescence (for neutrophil GFP expression, shown in green) and transmission (for zebrafish morphology, shown in grey) acquisitions. 3D rendering performed in Blender.
(MP4)

S2 Video 3D reconstruction from a full DoF OPT acquisition of a 4 dpf trangenic zebrafish at NA ~0.035. Red channel—white light transmission measurements, contrast is given through absorption. Green channel—GFP expression in neutrophils. Visualisation and rendering produced in Blender (Blender Ltd). Initial width ~586 μm, reducing to equivalent region of interest of ~130 μm.
(MP4)

S3 Video 3D reconstruction from a RoI-OPT acquisition of a 4 dpf trangenic zebrafish with a SR of ~130 μm at NA ~0.4. Red channel—white light transmission measurements, contrast is given through absorption. Green channel—GFP expression in neutrophils. Visualisation and rendering produced in Blender (Blender Ltd). Initial width ~586 μm, reducing to equivalent region of interest of ~130 μm.
(MP4)

S4 Video Comparison between the different OPT approaches, imaging a suspension of 200 nm fluorescent microspheres. (a) Full DoF OPT, NA ~0.035. (b) Half DoF OPT, NA ~0.05. (c) RFS-OPT at maximum SR of ~400 μm and NA ~0.4. (d) RoI-OPT with a SR ~65 μm and NA ~0.4.
(MP4)

S5 Video 3D reconstruction from a half DoF OPT acquisition of a suspension of fluorescent microspheres at NA ~0.05. Visualisation and rendering produced in Blender (Blender Ltd). Initial width ~505 μm, reducing to equivalent region of interest of ~65 μm.
(MP4)

S6 Video 3D reconstruction from a RoI-OPT acquisition of a suspension of fluorescent microspheres with a SR of ~65 μm at NA ~0.4. Visualisation and rendering produced in Blender (Blender Ltd). Initial width ~505 μm, reducing to equivalent region of interest of ~65 μm.
(MP4)
