# Supplementary material for: OPTiM: Optical projection tomography integrated microscope using open-source hardware and software
Source: PLoS One. 2017 Jul 11;12(7):e0180309. doi: 10.1371/journal.pone.0180309 (PMC5507440; doi:10.1371/journal.pone.0180309)
Supplement: S1 File — (DOCX) [file pone.0180309.s001.docx]

**Open source adaptation of a commercial inverted microscope for inexpensive in vivo 3D imaging by Optical Projection Tomography – Supplementary Material**

Thomas Watson^1^, Natalie Andrews^1,2^, Samuel Davis^1^, Laurence Bugeon^2^, Margaret D. Dallman^2^ and James McGinty^1^ Photonics Group, Department of Physics, Imperial College London, SW7 2AZ, UK

^2^ Department of Life Sciences, Imperial College London, SW7 2AZ, UK

**OPT Adaptor Plate**

The OPT adaptor was designed to fit in a common 160 × 110 mm microscope stage aperture and consists of three main aluminium fabricated components: the base plate that sits securely in the microscope stage aperture, the top plate that can be tilted with respect to the base and the sample chamber itself, attached to the top plate and in which the sample is mounted in a refractive index matching liquid (e.g. water for *in vivo* imaging).

S1 Fig: CAD rendering of the OTP adaptor. View of (a) the top plate, (b) the bottom plate and (c) sample chamber, stepper motor and axle adaptor in which the tube containing the sample is inserted and held.

S1 Fig shows three views of a CAD rendering of the OPT adaptor. To provide controlled adjustment of the tilt angle, the two plates are separated at one end by a 6 mm diameter and 60 mm long aluminium dowel seated in grooves on each plate and a fine thread adjustment screw (P25SB100L, Thorlabs Inc) at the other. The plates are held together using four 12 mm tension springs and retaining pins at each corner. The stepper motor (NM08AS-T4, Laser 2000 UK Ltd) is mounted directly on the side of the sample chamber, with its axle passing through a rubber O-ring in the chamber wall. An aluminium mounting port is attached to the motor axle to allow the FEP tubing (containing the sample) to be easily mounted in the chamber. The chamber base has an aperture over which a 22 mm diameter coverslip can be waxed into position. The full CAD files for the OPT adaptor can be downloaded from <http://www.imperial.ac.uk/photonics/research/biophotonics/instruments--software/optical-projection-tomography-opt/opt-microscope-adaptor-plate/>. The dimensions of the adaptor can be adjusted to fit onto different microscope stages (e.g. well plate aperture) and the design adapted for different stepper motors, FEP tubing diameters, etc.

**Calibration and Alignment**

As a user becomes more experienced in performing OPT, the alignment procedure can be reduced to comparing two images acquired at 180° relative rotation, since for an ideally aligned system the two images should precisely overlap when one of the images is flipped about its centre. Here we describe a more comprehensive alignment and calibration procedure using a sample comprised of a low concentration of fluorescent beads (we typically use 200 nm fluorescent microspheres – F8848, Thermofisher Scientific Inc).

*Fluorescent beads sample preparation*

1. Identify sub-resolution diameter fluorescent beads with similar excitation/emission characteristics to your biological sample. If you are using the beads for the calibrations procedure (not resolution measurements) you can use beads that are up to ~20x larger for increased fluorescence signal.
2. Prepare a 1% solution of low melting point agarose.
3. While the agarose solution is still liquid, mix the beads with the agarose at desired concentration (~20/mm^3). Image a small volume of the solution on a coverslip to assess if concentration is appropriate.
4. Insert blunt needle into a length of FEP tubing (~10-15 cm).
5. Using a syringe, draw the bead suspension solution through the blunt needle into the FEP tubing.
6. Remove needle and allow agarose to cool and gel.
7. Cut the tubing into appropriate lengths and store in water and shield from light. These samples will last several weeks.

S2 Fig: Diagram of calibration procedure for real data of fluorescent microspheres (200nm diameter), 4x magnification using system from fig 1c. (a) Raw projection from full-DoF OPT acquisition. (b) The spheres are found from the raw projections, and their trajectories recorded using simple peak finding MATLAB software (colour represents the same sphere through figs b-f). (c) The mean value of the recorded x-positions provides the axis of rotation (AoR) horizontal shift, δ from sensor centre, for each z-position. Applying a linear fit, gives the AoR rotation angle ζ. These values are used before reconstruction to shift and rotate the raw projections. (d) Sinusoid fitted to the recorded x-positions for all sphere traces. (e) Recorded y-positions of the microspheres. If all spheres have the same amplitude of variation over the acquisition cycle, this suggest there exists a tilt in the system, φ, and the custom stage angle will need to be altered. If the spheres y-deviation varies across the FoV, this suggest the system is not telecentric, and an external aperture and relay system may be required (only necessary for >4x magnification). (f) Difference between recorded x-positions and fitted sinusoid. Compare deviations in (e-f) with the diffraction limit of this system is ~4.5µm / 3 pixels (reduced NA~.055). As these effects are significantly smaller than the diffraction limit, the impact on reconstruction quality will be negligible.

*Calibration and acquisition procedure for direct OPT (low magnification)*

1. Using the full NA of the objective, focus to the axis of rotation (AoR). This is achieved by continually refocussing onto a bead while it rotates until it is at a lateral extreme of its motion and therefore in the same plane as the AoR. After this do not re-focus the microscope (see S2(a) Fig).
2. Reduce the NA of the objective using an appropriate aperture so that the depth of field covers the full extent of the sample (i.e. full depth of field OPT).
3. Perform a full acquisition on the fluorescent bead sample (e.g. 400 images at 0.9° steps).
4. Display the sum of all the projection images (or maximum projection across all images).
5. If the bead traces form straight lines (not elliptical paths) then the system is telecentric and has no axial tilt (i.e. ϕ ≈ 0°, see S2(b) Fig). Go to #11.
6. If the bead paths are elliptical, apply a basic segmentation algorithm to each projection image and extract the (x,y) coordinates of each bead (i.e. as a function of rotation angle).
7. Reorder these coordinates so that they all start with their minimum x-position and plot (x,y-y_0_) for every bead up to 180° (i.e. the first half of the reordered data), where y­_0_ is the first y-value in the reordered coordinates for a bead.
8. If the sign of all bead traces are the same (i.e. all plots have either y-y_0_≥0 or y-y_0_≤0), then there is a significant tilt (i.e. ϕ ≠ 0°). Iteratively apply a correction to the adaptor plate and reacquire the bead data to correct.
9. After correction, if the bead traces form straight lines (not elliptical paths) then the system is telecentric and has no axial tilt (i.e. ϕ ≈ 0°, see S2(b) Fig). Go to #11.
10. If the sign of the bead traces are **not** the same (i.e. plots have both y-y_0_≥0 and y-y_0_≤0), then the system is non‑telecentric and an image relay is required to position the aperture in a conjugate plane.
11. The angle of these straight lines with respect to the rows of pixels is the AoR rotation angle ζ. If this angle is large rotate the camera and repeat the acquisition until ζ ≈ 0°.
12. With ζ ≈ 0°, the common centre of the bead traces is the AoR. Any relative displacement between this AoR and the centre of the image (δ) can be corrected by translating the microscope stage or in pre-processing before reconstruction. Combined analysis for ζ and δ is shown in S2(c) Fig.
13. For a correctly aligned system (including post-acquisition correction for ζ and δ if required) the segmented x-position for each bead should follow a sinusoidal path (see S2(d) Fig). Deviations from sinusoidal motion and constant y‑position that are below the resolution limit (S2(e,f) Figs) are tolerable and the data can be directly reconstructed with no further correction.

*Calibration and acquisition procedure for OPT with external relay*

1. For conventional OPT or RFS-OPT, set the iris to its maximum diameter (or remove aperture) and ETL current to the mid‑point value respectively. Focus to the AoR (same approach as outlined previously).
2. Reduce the iris diameter (or insert aperture) or increase the current limits of the ETL until the whole sample is within the DoF or scan range, for conventional OPT and RFS respectively.
3. Perform a full acquisition on the fluorescent bead sample (e.g. 400 images at 0.9° steps).
4. Perform the same analysis outlined previously in #4-9.
5. If non‑telecentric performance is observed, iteratively apply a correction to the iris/aperture/ETL axial position and reacquire bead data to correct.
6. Once corrected for telecentricity, go to #11-12 for final alignment correction.

S3 Fig: Data acquired using 20x objective, in an external relay setup. (a) Example of a bean sinogram of a 4 µm fluorescent microsphere with associated fitted sinusoid (dotted line). (b) Measured AoR motion for different acquisition modes. Note the additional RFS trace represents a second independent acquisition demonstrating the repeatability of the motion. Single slice reconstructions of 4 µm fluorescent microspheres (c) without and (d) with motor motion correction applied.

*Calibration of stepper motor motion precession*

At higher magnifications lateral movement of the AoR may be observed, caused by precession of the stepper motor axle during rotation. For the stepper motors we observe that this motion is repeatable and dependent on the absolute motor rotation position. Having performed the alignment procedure outlined above, this motion can be calibrated and corrected before reconstruction.

1. Under full depth of field OPT conditions, perform a full acquisition on the fluorescent bead sample (e.g. 400 images at 0.9° steps).
2. Apply the same rotation and shift correction to all images if required (i.e. if ζ ≠ 0° and/or δ ≠ 0).
3. Apply a basic segmentation algorithm to each corrected projection image and extract the (x,y) coordinates of each bead (i.e. as a function of rotation angle).
4. Fit sinusoids to the x-positions as a function of rotation angle for all bead traces (i.e. the sinogram for each bead). The difference between x-position and fitted sinusoid details the deviation of a bead from its expected trajectory (see S3(a) Fig).
5. If all the deviations are similar, there is a common motion caused by axle precession. We have found this to be a repeatable effect that is dependent on the absolute motor rotation position (see S3(b) Fig).
6. If this motor motion exceeds the diffraction limit of the system, the data can be corrected by applying the necessary shift to each projection image before reconstruction (compare S3(c,d) Figs).

**Effective Optical Transfer Function and Focal Scanning OPT Spatial Resolution**

In the main paper we presented the effective PSF (ePSF) for RFS- and RoI-OPT, which is given at every z-plane by the integral of the intrinsic PSF over the scan range weighted by the relative dwell time per focal position, W(z) (determined by the functional form of the axial scan),

$ePSF\left(x,y,z\right)=\int_{SR} W\left(z'\right)PSF\left(x,y,z'+z\right)dz'$.

(1)

S4 Fig: Example of change in optical transfer function (OTF), when implanting focal scanning with different scan ranges (SR). Simulations modelled in MATLAB for 20x, 0.4NA objective, focusing into 3mm of water. (a) 2D representation OTF of RFS-OPT with a scan range of ~65µm (40mA current modulation). (b) RFS-OPT at maximum scan range of ~450µm (290mA current modulation). (c) Example of static OTFs at increasing focus depths. (d) Line profiles across centre of OTF, plotted on log scale, normalized to 1. Spatial frequency is normalized to the cut-off frequency for an NA of 0.4. Also shown are the diffraction limited (DL) profiles for an NA~0.4, which is the full NA used in the scanning procedure, and a reduced NA~0.03, which is equivalent to a depth of field ~450µm.

To appreciate how varying the scan range effects the achievable spatial resolution in the final reconstruction through the contrast-to-noise ratio, it is more informative to consider the effective optical transfer function (the lateral Fourier Transform of the ePSF), which is similarly given by the axial integration of the intrinsic OTF,

$$eOTF\left(u,v,z\right)=\int_{SR} W\left(z'\right)OTF\left(u,v,z'+z\right)dz'$$

(2)

S4(a,b) Figs show the eOTF for a scan range of ~65 and ~450 µm respectively, simulated for a ×20 0.4 NA objective lens scanned linearly. S4(d) Fig shows the eOTF at a particular z-plane for these two scan ranges (green and red respectively) as well as the intrinsic in-focus OTF (black). Consider an object in this z-plane. During the acquisition of a projection the relative amount of time it is imaged in-focus is given by W(z). Therefore the shorter the scan range the greater the relative contribution the high spatial frequency components make to the projection signal, increasingly the probability that these frequencies will be measured above the noise floor and contribute to the reconstruction, as demonstrated in the next section.

**Comparison of external relay systems using fluorescent microspheres**

To determine the relative performance of the different OPT approaches, phantoms consisting of fluorescent microspheres were imaged. 200 nm diameter microspheres (F8848, Thermo Fisher Scientific Inc) were suspended in agarose at a concentration that emulated a transgenic zebrafish embryo (i.e. similar fluorescent signal measured using similar acquisition settings). Projections were acquired at a fixed integration time of 0.1 s per frame, with 400 projections taken over 360 (i.e. every 0.9°) resulting in a total acquisition time of ~110 s. Conventional full and half DoF OPT corresponded to an effective NA of ~0.035 and ~0.05 respectively. RFS-OPT acquisitions were performed at maximum scan range of ~400 µm, while RoI-OPT followed a cylindrical region of interest with a scan range ~65 µm. The ETL was operated with a sinusoidal modulation at 10 Hz. The raw projection data acquired from these different OPT approaches are shown in S5 Fig and S4 Video.

S5 Fig: Comparison between the different OPT techniques, looking at a raw projection of >200nm fluorescent microspheres. (a) Conventional OPT with the DOF covering the whole sample, NA~0.025. (b) Conventional OPT with the DOF covering the front half of the sample. (c) RFS-OPT at maximum scan range. (d) RoI-OPT tracking a region of width ~65µm. The dashed-red circle represents the object used to perform the pre-scan procedure, and lies with the region of interest for RoI-OPT.

Resolution measurements were taken from a representative subset of the smallest objects reconstructed and fitted to a Gaussian function (S6(a‑c) Figs). Conventional half DoF OPT gave an experimental resolution of 5.6 ± 0.4 µm. This was improved by RFS-OPT to 2.2 ± 0.3 µm and by RoI-OPT to 1.6 ± 0.2 µm (see S6(e) Fig). To quantify the increase in acquired signal for the scanning approaches, the CNR between the same object and the local background level were calculated for each acquisition approach [Welvaert, M. and Rosseel, Y. “On the definition of signal-to-noise ratio and contrast- to-noise ratio for fmri data”. PLoS ONE, 8(11):e77089, 2013]. The CNR ratio for half DoF OPT was 0.6 ± 0.8. For RFS-OPT this increased to 1.8 ± 1.3, while for RoI-OPT it further increased to 11.4 ± 3.4.

As can be seen in S6(g) Fig, objects located outside the scan range appears distorted in the reconstruction due to being imaged both inside and outside the SR over the complete acquisition cycle (i.e. in and out of focus). A similar effect can be observed in conventional OPT if the DoF is too small. Note that the non-linear colour scale applied exaggerates the appearance of this distortion. Comparisons between the conventional half DoF OPT and RoI-OPT reconstructions are available in S5 Video and S6 Video respectively. RoI-OPT can additionally reveal objects that are not visible in conventional or RFS-OPT images, through the suppression of streak artefacts created by bright object outside the region of interest (see S6(i-k) Figs).

S6 Fig: Cross sections (CS) through reconstructed volume of 200nm fluorescent microspheres. (a) Resolution measurements on reconstructed slices of fluorescent microsphere sample. Note the spheres depicted in (a-c) are not the same, but are representative of the smallest object reconstructed. (a) Half DoF OPT, NA ~0.05 (full DoF OPT not represented as the resolution was significantly worse). (b) RFS-OPT with ~400 µm scan range and NA ~0.4. (c) RoI-OPT with scan range reduced to ~65 µm and NA ~0.4. (d) Gaussian fits and raw data from the line profiles shown in (a-c) showing increase in light collection efficiency. (e) Normalised gaussian fitting to illustrate resolution improvement from conventional to RFS systems. (f-h) Reconstructed slice through a sample of 200 nm fluorescent microspheres for (f) Half-DoF OPT (and Media 5), (g) RFS-OPT and (h) RoI‑OPT (and Media 6). ETL scan range shown by red circle. Note that colour scales are non-linear to display both bright and faint objects. (i-k) Magnified view of region of interest (yellow box), highlighting the improvement in CNR and reduction of streak artefacts within the region of interest with RoI-OPT (with linear colour scales).
