# Supplementary figures and images for: OPTiM: Optical projection tomography integrated microscope using open-source hardware and software
Source: PLoS One. 2017 Jul 11;12(7):e0180309. doi: 10.1371/journal.pone.0180309 (PMC5507440; doi:10.1371/journal.pone.0180309)

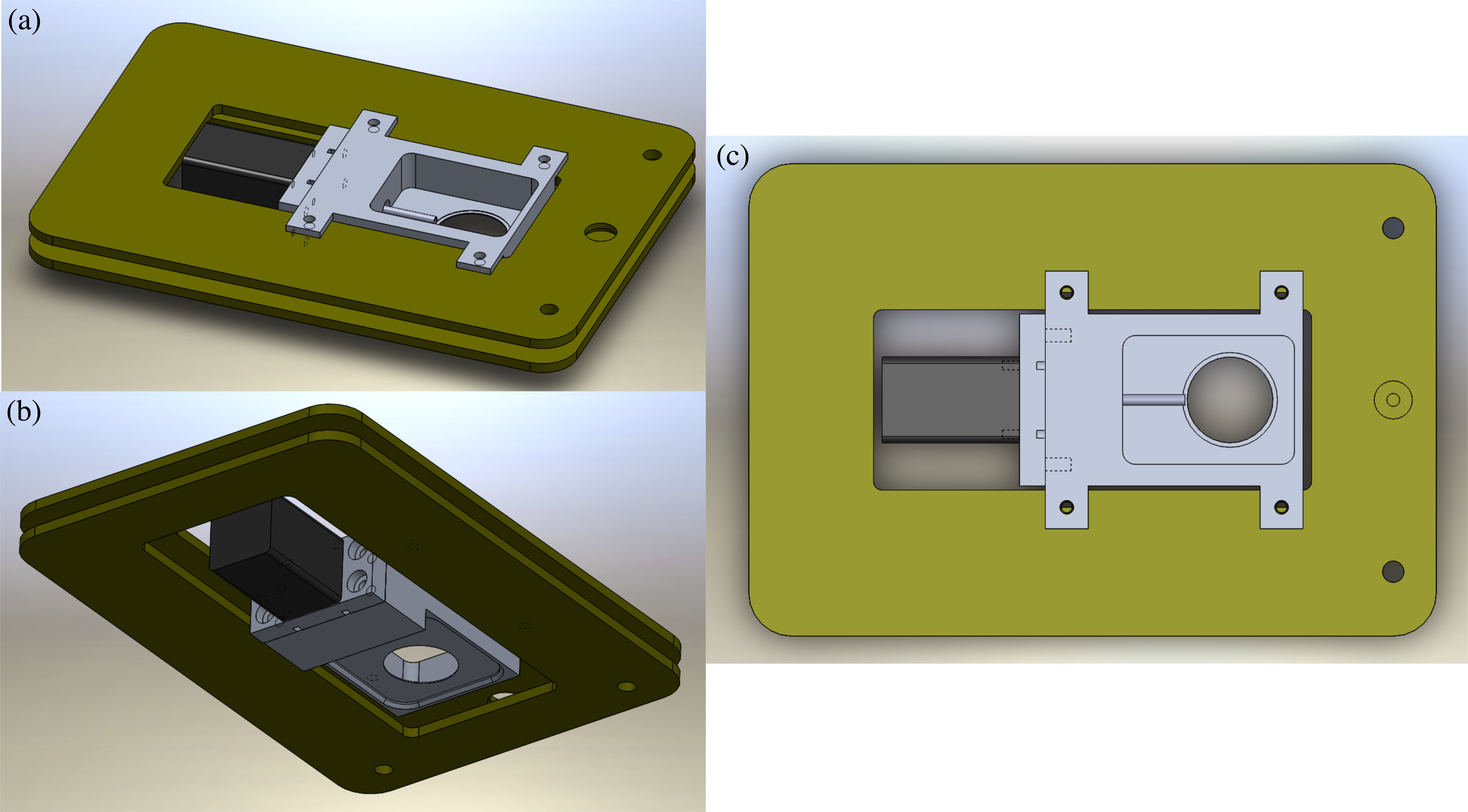

Supplement: S1 Fig — View of (a) the top plate, (b) the bottom plate and (c) sample chamber, stepper motor and axle adaptor in which the tube containing the sample is inserted and held. (TIFF) [file pone.0180309.s002.tiff]

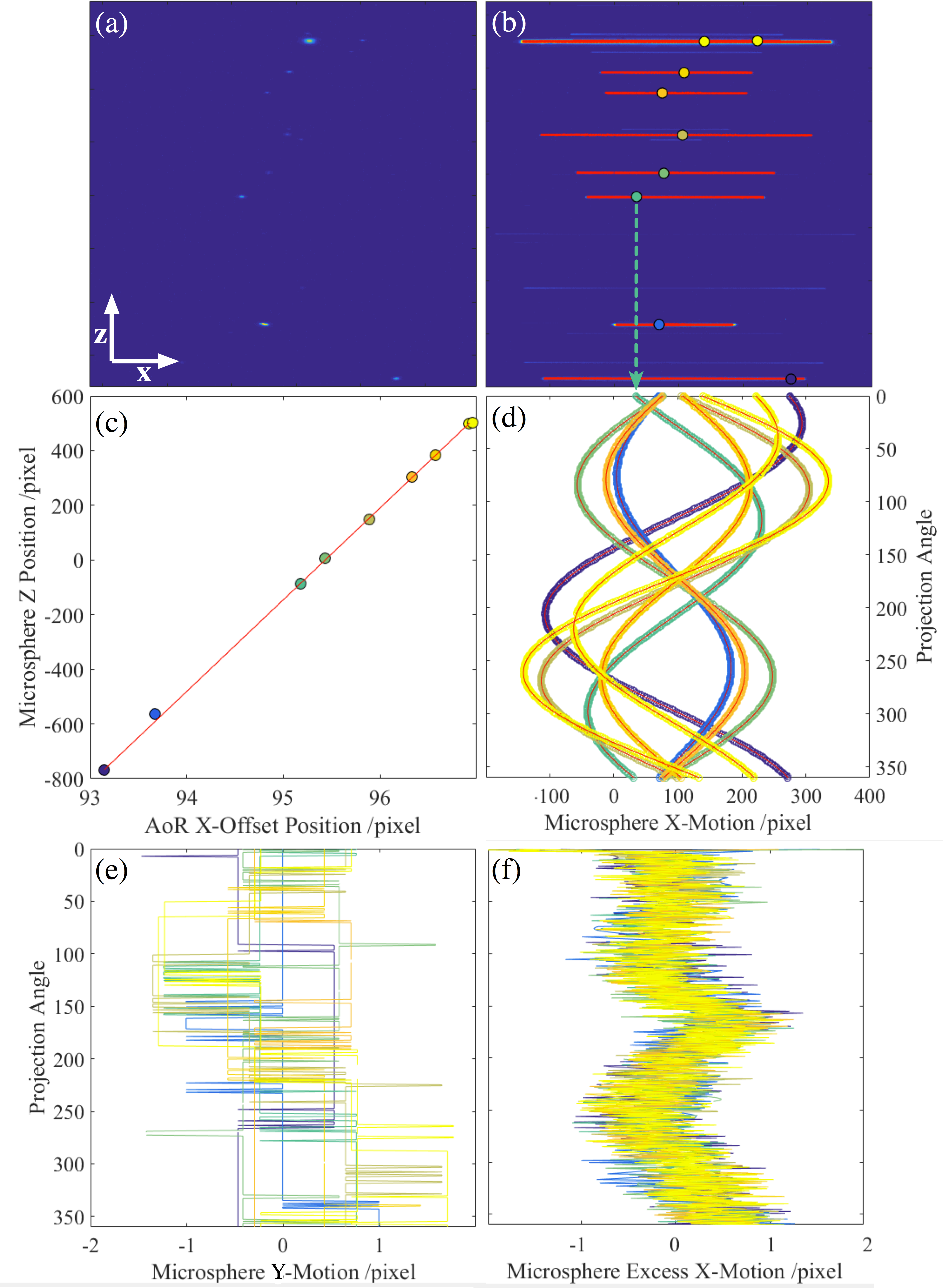

Supplement: S2 Fig — (a) Raw projection from full-DoF OPT acquisition. (b) The spheres are found from the raw projections, and their trajectories recorded using simple peak finding MATLAB software (colour represents the same sphere through figs b-f). (c) The mean value of the recorded x-positions provides the axis of rotation (AoR) horizontal shift, δ from sensor centre, for each z-position. Applying a linear fit, gives the AoR rotation angle ζ. These values are used before reconstruction to shift and rotate the raw projections. (d) Sinusoid fitted to the recorded x-positions for all sphere traces. (e) Recorded y-positions of the microspheres. If all spheres have the same amplitude of variation over the acquisition cycle, this suggest there exists a tilt in the system, φ, and the custom stage angle will need to be altered. If the spheres y-deviation varies across the FoV, this suggest the system is not telecentric, and an external aperture and relay system may be required (only necessary for >4x magnification). (f) Difference between recorded x-positions and fitted sinusoid. Compare deviations in (e-f) with the diffraction limit of this system is ~4.5μm / 3 pixels (reduced NA~.055). As these effects are significantly smaller than the diffraction limit, the impact on reconstruction quality will be negligible. (TIFF) [file pone.0180309.s003.tiff]

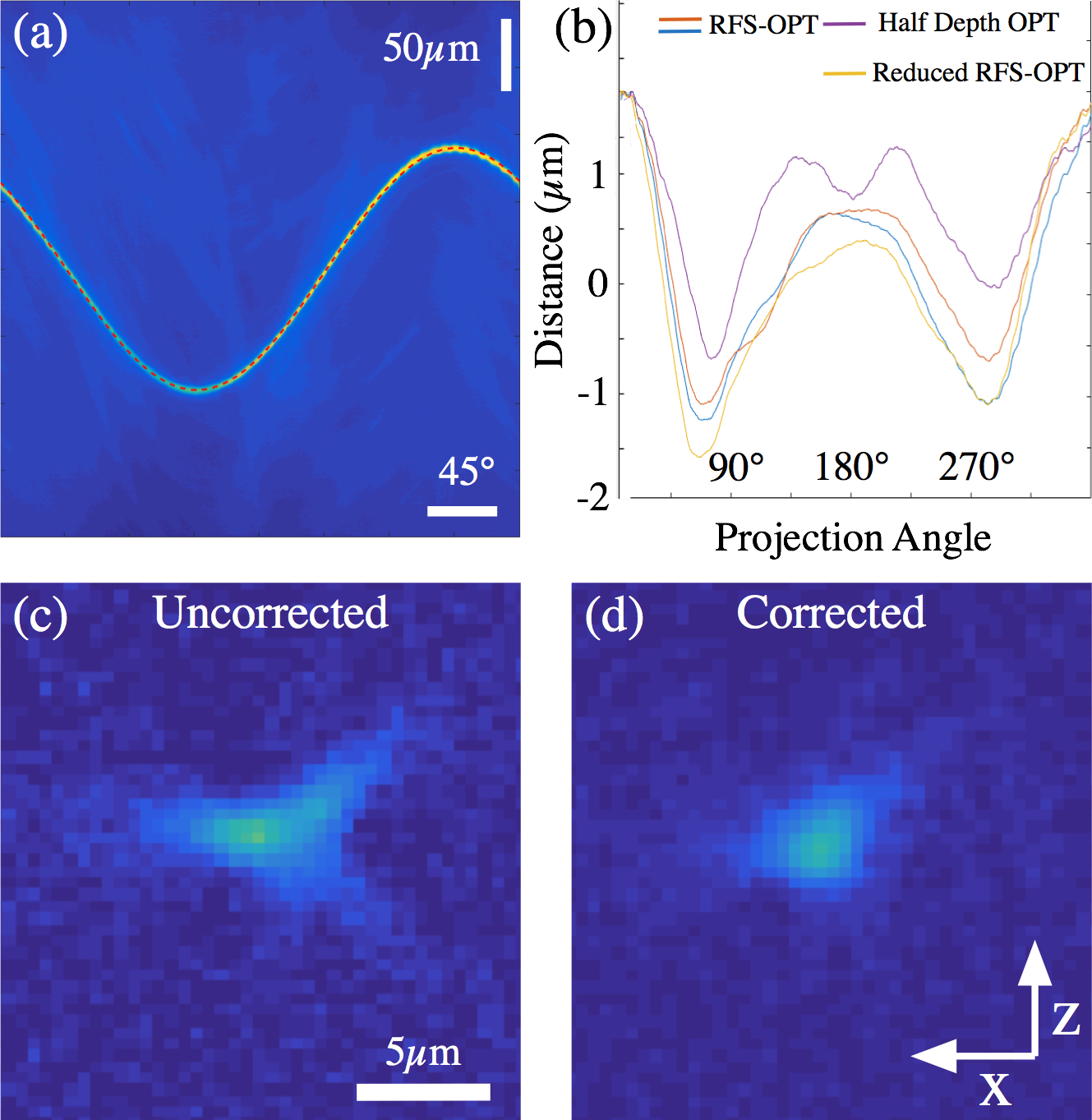

Supplement: S3 Fig — (a) Example of a bean sinogram of a 4 μm fluorescent microsphere with associated fitted sinusoid (dotted line). (b) Measured AoR motion for different acquisition modes. Note the additional RFS trace represents a second independent acquisition demonstrating the repeatability of the motion. Single slice reconstructions of 4 μm fluorescent microspheres (c) without and (d) with motor motion correction applied. (TIFF) [file pone.0180309.s004.tiff]

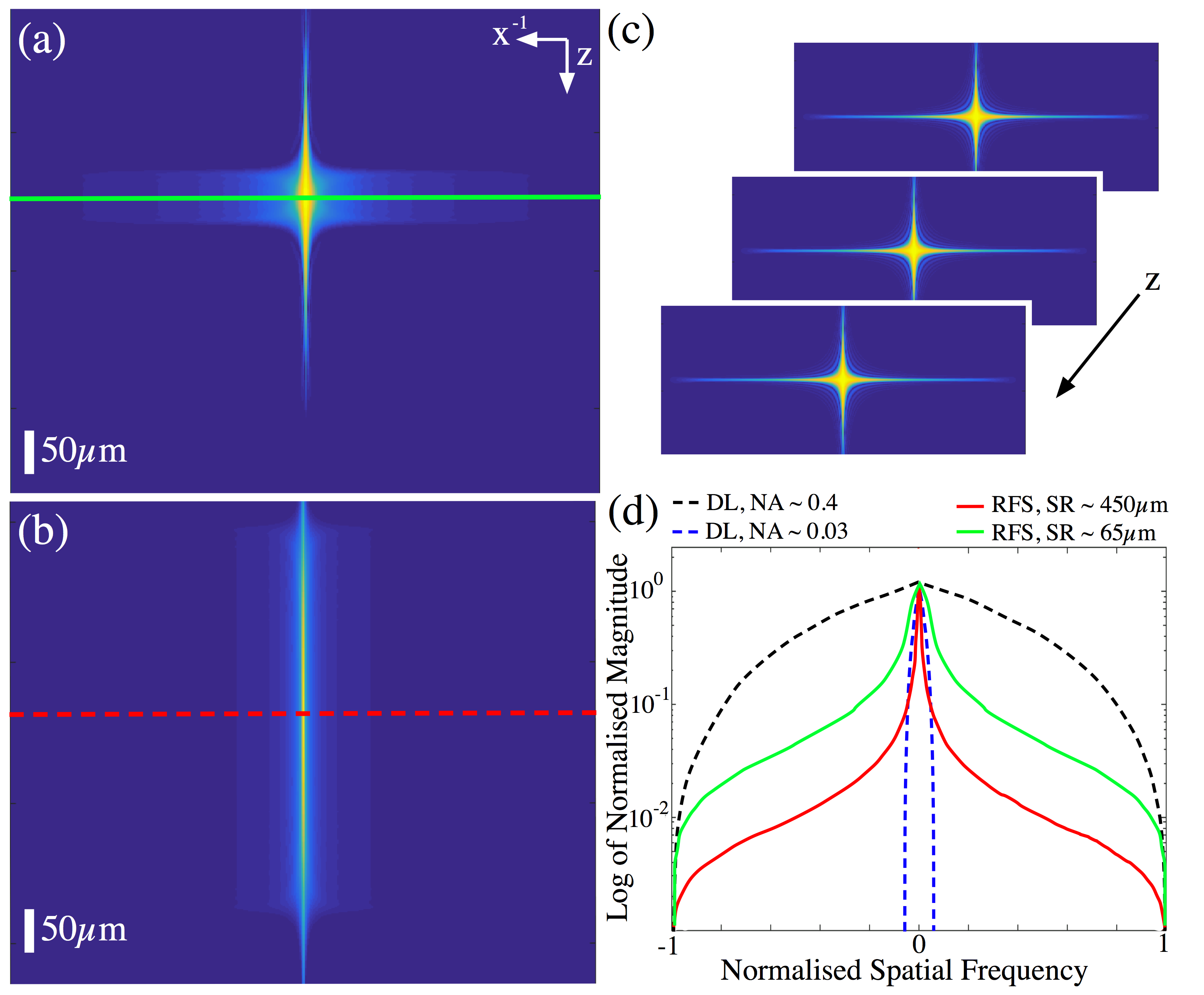

Supplement: S4 Fig — Simulations modelled in MATLAB for 20x, 0.4NA objective, focusing into 3mm of water. (a) 2D representation OTF of RFS-OPT with a scan range of ~65μm (40mA current modulation). (b) RFS-OPT at maximum scan range of ~450μm (290mA current modulation). (c) Example of static OTFs at increasing focus depths. (d) Line profiles across centre of OTF, plotted on log scale, normalized to integrate to 1. Spatial frequency is normalized to the cut-off frequency for an NA of 0.4. Also shown are the diffraction limited (DL) profiles for an NA~0.4, which is the full NA used in the scanning procedure, and a reduced NA~0.03, which is equivalent to a depth of field ~450μm. (TIFF) [file pone.0180309.s005.tiff]

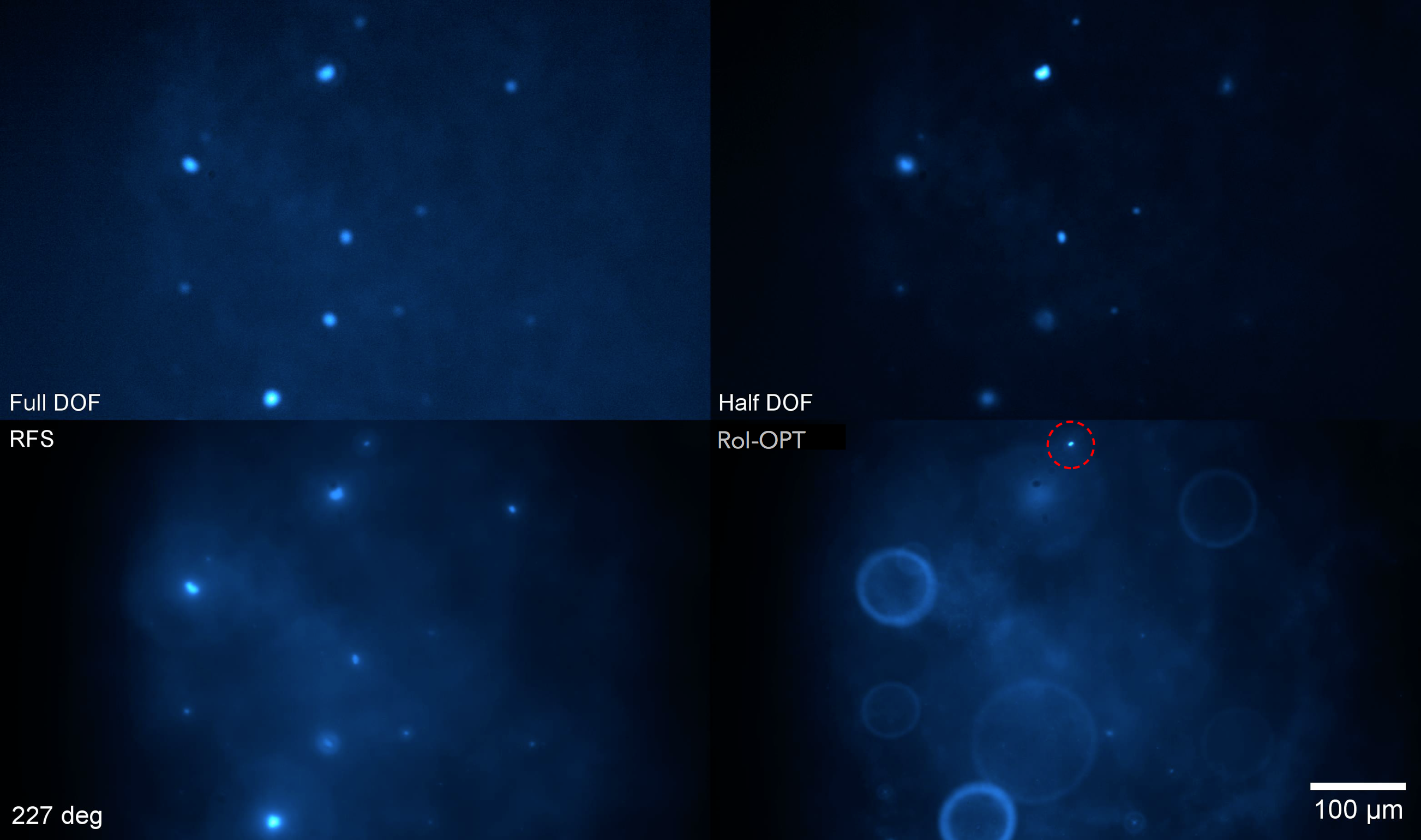

Supplement: S5 Fig — (a) Conventional OPT with the DOF covering the whole sample, NA~0.025. (b) Conventional OPT with the DOF covering the front half of the sample. (c) RFS-OPT at maximum scan range. (d) RoI-OPT tracking a region of width ~65μm. The dashed-red circle represents the object used to perform the pre-scan procedure, and lies with the region of interest for RoI-OPT. (TIFF) [file pone.0180309.s006.tiff]

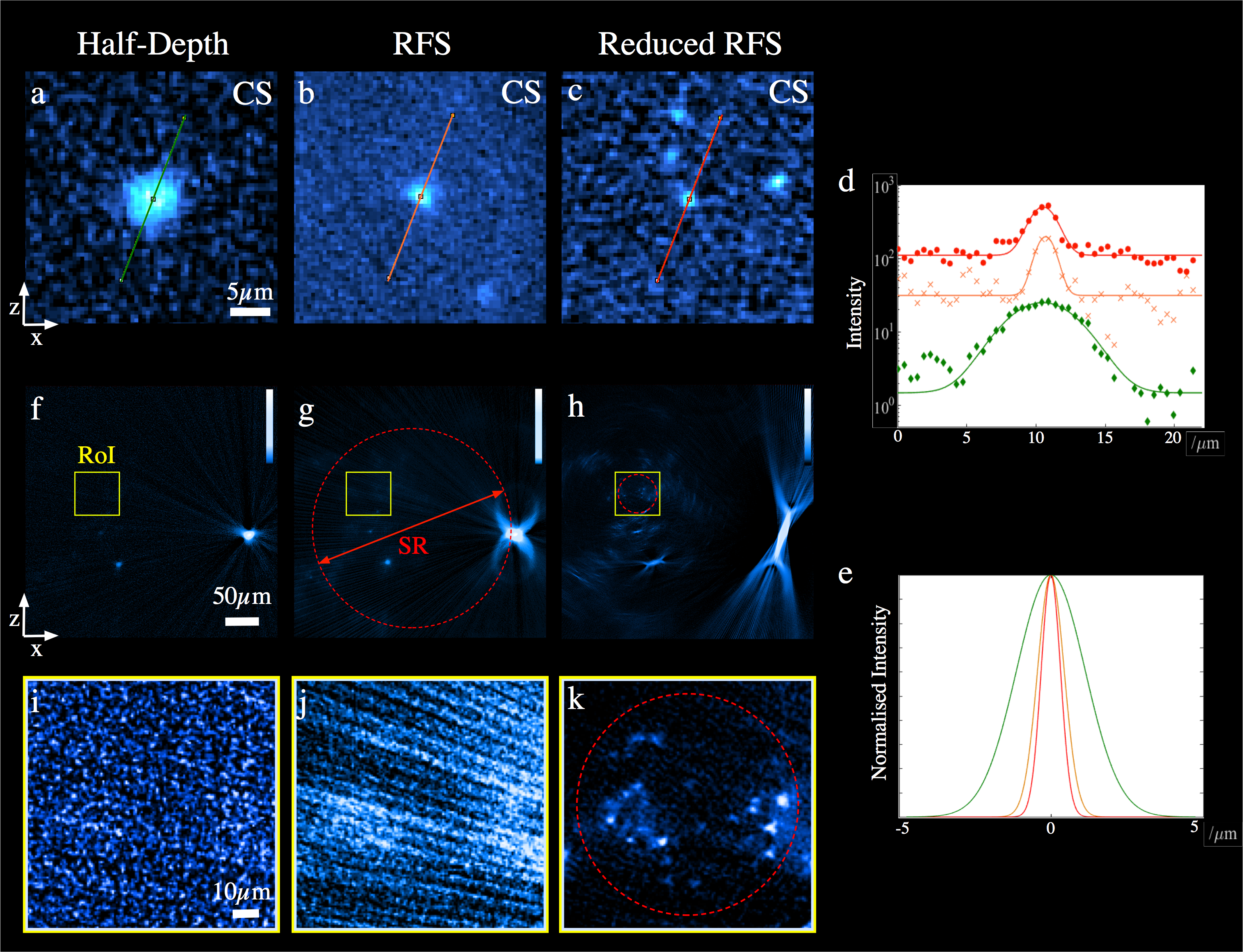

Supplement: S6 Fig — (a) Resolution measurements on reconstructed slices of fluorescent microsphere sample. Note the spheres depicted in (a-c) are not the same, but are representative of the smallest object reconstructed. (a) Half DoF OPT, NA ~0.05 (full DoF OPT not represented as the resolution was significantly worse). (b) RFS-OPT with ~400 μm scan range and NA ~0.4. (c) RoI-OPT with scan range reduced to ~65 μm and NA ~0.4. (d) Gaussian fits and raw data from the line profiles shown in (a-c) showing increase in light collection efficiency. (e) Normalised gaussian fitting to illustrate resolution improvement from conventional to RFS systems. (f-h) Reconstructed slice through a sample of 200 nm fluorescent microspheres for (f) Half-DoF OPT (and Media 5), (g) RFS-OPT and (h) RoI‑OPT (and Media 6). ETL scan range shown by red circle. Note that colour scales are non-linear to display both bright and faint objects. (i-k) Magnified view of region of interest (yellow box), highlighting the improvement in CNR and reduction of streak artefacts within the region of interest with RoI-OPT (with linear colour scales). (TIFF) [file pone.0180309.s007.tiff]
